# An Augmented Risk Information Seeking Model: Perceived Food Safety Risk Related to Food Recalls

**DOI:** 10.3390/ijerph15091800

**Published:** 2018-08-21

**Authors:** Chuanhui Liao, Xiaomei Zhou, Dingtao Zhao

**Affiliations:** 1 School of Economics and Management, Southwest University of Science and Technology, Mianyang 621010, China; Zhouxiaomei195@163.com; 2 School of Management, University of Science and Technology of China, Hefei 230026, China

**Keywords:** current knowledge, food recall, perceived information gathering capacity, risk communication, risk perception

## Abstract

Food safety is of worldwide concern. As an effective mechanism governing food safety, food recalls are widely applied around the world. Though it is well known that food recalls can have substantial, negative impacts on corporate reputation and marketing, we know relatively little related to what factors motivate people to seek related information after the recall announcement. This study attempts to elucidate the determinants of information-seeking intention in the context of food safety in food recalls by using an augmented risk information-seeking model. A survey of 631 Chinese residents was used to explore the proposed framework. The results show that current knowledge, risk perception, perceived channel beliefs, and perceived information-gathering capacity (PIGC) are all significant predictors of information need and information-seeking intention. It was also confirmed that risk perception has a positive correlation with seeking need. These findings are important for policymakers, recalling manufacturers, and retailers to develop strategies for better risk communication in food recall announcements.

## 1. Introduction 

Food safety is a worldwide concern and a threat to sustainable production and consumption. According to WHO, 31 foodborne hazards caused 600 million foodborne illnesses and 420,000 deaths in 2010 [1]. With repeated major food incidents occurring in China, food and drug safety issues are listed as top interests of the Chinese government and the public [2]. As a well-established and effective system, food recall has been applied worldwide. In a survey of 1101 respondents conducted in 50 American states in 2008, most of the participants viewed food recalls as an important measure to save lives [3]. A food recall is also a major concern for the stakeholders in the food industry chain because responses to a food scare arising from a food recall can cause significant economic effects and reputation losses to the recalling firms, the supply chain, and even the whole industry with spillover effects [4,5]. In China, the food recall system was established in 2007 when the General Administration of Quality Supervision (Inspection and Quarantine Ministry of the People’s Republic of China) issued the “Regulations on the Management of Food Recalls” [6]. Then, late in 2015, the “Measures for the Management of Food Recalls” [7] was issued by the China Food and Drug Administration (CFDA) to make the process more practical. In these regulations and measures, a food recall can either be a voluntary one initiated by the recalling manufacturers and retailers, or a mandatory one required by the CFDA and its subordinates at the county level or above. Since the implementation of these regulations and measures, food recall has become a major consideration in the management of food safety issues [8]. 

As a necessity, food is ubiquitous and so everyone is affected by defective food products [2]. Even if few people consumed the defective food, some major food recalls may cause food scares in the public [9]. After a major food recall, people may perceive higher food risk with food safety and lower trust in the administrative management [4]. Though most U.S. residents believe that it is extremely important to learn about recall information and related knowledge, they still fail to utilize existing resources to gain adequate and accurate information to make response action decisions about recalled products [3]. Hence, the U.S. Government Accountability Office (GAO) initiated a proposal for better industry information sharing after reviewing the 2003 outbreak of mad cow disease. Timely, adequate, and accurate information is the focus of food recall communication, and it is also an important factor influencing residents’ behavioral decisions when they face food recalls and food safety issues.

In risky situations of food safety incidents, the consumer’s behavior is often influenced more by psychological factors than the physical properties of the food products concerned [10]. Perception of risk is always associated with major food incidents, which results in a negative effect (worry) [11]. Then people may recognize information insufficiency and may seek additional information to assess the certainty, severity, and immediacy of the risk [12]. They can use traditional mass media, such as radio, television, peers, and newspaper or they can employ internet-based social media. Various models have been developed to answer these questions, from Griffin’s Risk Information-Seeking and Processing model (RISP) [11] to Kahlor’s Planned Risk Information-Seeking Model (PRISM) [13]. These models had been employed in different situations such as health risk, flood risk, and global warming [14,15,16]. Although Griffin et al. [17] indicated that RISP is more appropriate for research of relatively familiar situations or those personally relevant to respondents, to the best of our knowledge, few studies have employed these type of model in the context of food recalls. In the domain of food safety and food recall, the literature has elaborated on economic, social, and technical effects of food recalls [18,19], while less concern has been paid to consumers’ psychological and behavioral processes concerned with their perceptions of food safety in food recalls [20]. Of all the stakeholders involved, the consumers’ reactions are what leads to diminishing sales and loss of reputation. Learning how the consumers react to food recall announcements, why and how they evaluate and seek information, and how they make purchasing decisions, is what firms and policymakers need to know to develop appropriate strategies and policies to mitigate the negative effects and to perfect the recall system [21]. Since research in this domain is still limited [19], this paper intends to explore consumers’ psychological and behavioral reactions to food recall announcements, focusing on the determinants of the information-seeking decision.

There are many reasons for food recalls. Reasons for food recalls in China are quite different from those in Western countries. In the West, most of the major food incidents and food recalls arise from natural bacteria [22] but in China, food additives have become the most common causes of food safety incidents and a major public concern [23]. Here, we specifically define the *abuse of food additives* as illegal use of a legal additive and intentional addition of illicit substances into food. The reason that there are less food safety incidents caused by natural bacteria is mainly attributable to the Chinese dietary culture, in which most of the food is cooked, since most natural bacteria will die under high temperature. According to the Food Safety Risk Assessment Report of Shenzhen released by the Market and Quality Supervision Commission of Shenzhen Municipality, abuse of food additives is listed as the primary factor causing food safety problems in Shenzhen, a developed megacity in southern China. Also, the *Introduction to the 2017 China Development Report on Food Safety* [24] reported that food safety incidents arising from the illegal use of food additives and the intentional addition of nonfood substances into food account for 6% and 35% of the total incidents occurred from 2007 to 2016, respectively. Hence, in this study, we specifically test the influencing factors of information-seeking intention in the context of food recall related to abuse of food additives in China.

This paper is organized as follows: after the Introduction (Section 1), Section 2 presents the conceptual framework. In Section 3, the data and methodology are introduced, followed by data analysis and results in Section 4. Then a discussion is given in Section 5. Conclusions, implications, and comments on limitations are given in the last section (Section 6).

## 2. Conceptual Framework 

### 2.1. Models of Risk Information Seeking

Information-seeking refers to purposive seeking behavior for information to fulfill the need to satisfy some goal [25]. Based on the heuristic-systematic model (HSM) [26], risk research theory [27], media theory [28], and the theory of planned behavior [29], Griffin et al. [11] developed the RISP model. This model was initially proposed to explore how cognitive and socio-psychological variables contribute to individuals’ information-seeking and processing behaviors. It suggests that information-seeking and processing are primarily motivated by information insufficiency [30], which is the difference between current knowledge and the sufficiency threshold [13,31]. In addition to the motivations mentioned above, the RISP model alludes to information-seeking and processing through relevant channel beliefs (RCB) and perceived information-gathering capacity (PIGC). Relervant channel beliefs analyzes whether or not a particular information channel could provide useful, unbiased, and trustworthy information [11]. Individuals who hold a high perceived image of a specific media source will form a habitual information processing strategy and choose to seek information via this channel [28]. Perceived information-gathering capacity refers to the perceived ability to perform seeking and processing behavior, especially in a situation which requires cognitive effort and nonroutine gathering of information. Evidence indicates that PIGC plays a vital role in the determination of information-seeking and processing because people need to assess the various information options available and choose the most accessible channel [32]. Hence, the higher the level of PIGC an individual hold, the more perceived behavioral control he/she may possess to seek and process information. But according to Griffin et al. [14] and Yang et al. [32], most of the research work deals with the emergence of new research channels, whereas the direct effect of PIGC over information-seeking behavior remains at the exploratory stage due to a lack of consistency between conceptualization and operationalization.

After the RISP model, some researchers put forward extended and augmented models such as Kahlor’s [13] PRISM, Johnson and Meischke’s Comprehensive Model of Information Seeking [33], and Freimuth, Stein, and Kean’s Health Information Acquisition Model [34]. In PRISM, information-seeking is taken as a planned behavior, and the effects of individual-level variables (i.e., attitude toward seeking, seeking-related subjective norms, perceived seeking control, perceived current knowledge, and perceived knowledge insufficiency) are analyzed over the independent variable of information seeking intention. These risk information-seeking models have been proposed and applied in several situations but research on risk information-seeking in the context of food recalls remains scarce. Hence, in this paper, we investigate the direct and indirect effects of antecedent factors on information-seeking intention in the domain of food recall.

### 2.2. Research Model and Hypothesis Development

#### 2.2.1. Current Knowledge

Current knowledge refers to an individual’s estimation of his/her present amount of knowledge about the risk [11]. It appears as the first decision point for information-seeking in HIAM [35] since the primary purpose of information-seeking is to gain adequate knowledge to make a decision, and knowledge is regarded as a critical construct in information processing [36]. In the context of food safety, an individual’s knowledge base is predicted to be associated with information needs and information processing [36,37]. Though the findings in previous studies about the relationship between current knowledge and information need are contradictory, researchers generally agree that knowledge is a critical construct in information processing in the food industry [36]. Since the abuse of food additives is regarded as a dominant type of food safety risk, here we need to analyze the public’s knowledge about food additives. We propose that people with high levels of knowledge about what kind of additive is involved in a food recall will seek more knowledge and information they need to make a decision in a specific case. Similarly, Griffin [38] and Kahlor [16] proposed that individuals’ current knowledge about the specific risk would increase their capacity to gather new and additional knowledge. In other words, existing or current knowledge provides individuals with a “trained capacity” to gain access to and process additional information. Hence, the following hypotheses are proposed:

**Hypothesis 1a** **(H1a).** *Current knowledge is positively related to information need.*

**Hypothesis 1b** **(H1b).** *Current knowledge is positively related to information-seeking intention.*

**Hypothesis 1c** **(H1c).** *Current knowledge is positively related to PIGC.*

The relationship between current knowledge and risk perception has been well explored in the context of food safety. Generally, people tend to perceive less risk over familiar issues than those unfamiliar [39]. Those who have less objective knowledge (e.g., about chemical or technological processes in the food industry) attribute high risks to food products [39]. Previous studies have confirmed that when consumers have correct knowledge about gene technology in general, they perceive fewer risks in accepting genetic modification in food production [40,41]. Hence, the following hypothesis is developed:

**Hypothesis 1d** **(H1d).** *Current knowledge is negatively related to risk perception.*

#### 2.2.2. Risk Perception

Risk perception refers to “the assessment of how risky a situation is regarding probabilistic estimates of the degree of situational uncertainty, and confidence in these estimates” [42,43]. It is the central variable in the domain of risk information management. Both RISP [11,14] and PRISM [13] have confirmed the effect of risk perception on effective response, which leads to individuals’ need for information-seeking and processing. Griffin, Dunwoody, and Neuwirth [11] explored the effects of risk perception on information-seeking a decision in the context of mass media reports about a hazard. They found that perception of hazardous consequences produces greater information-seeking need and intention. The study of Huurne and Gutteling [44] focused on the determinants of information-seeking intentions in the context of treatment of hazardous industrial substances, and they found that risk perception is a direct predictor of intention to seek information. Therefore, the following hypotheses are considered in the current study:

**Hypothesis 2a** **(H2a).** *Risk perception is positively related to the information need.*

**Hypothesis 2b** **(H2b).** *Risk perception is positively related to information-seeking intention.*

#### 2.2.3. Information Need and Information Seeking Intention

In dealing with a risk issue, information need is defined as the gap between the knowledge held and perceived to be wanted [11]. It is also called information insufficiency in RISP [9] and PRISM [13]. It is the central factor in both RISP and PRISM and is predicted to be the motivation for information-seeking intention [32,43]. People are predicted to actively seek information under a high level of information need, especially in the circumstances involving personal issues such as food consumption and health risk [45,46,47]. In the domain of agriculture and food industry, Verbeke [48] revealed that the consumers’ perception of a real need for information is a conditional factor of information-seeking and processing. Moreover, the stronger the perceived need for information, the higher the probability that active seeking behavior will be performed [49]. Therefore, we propose the following hypothesis:

**Hypothesis 3** **(H3).** *Information need is positively related to information-seeking intention.*

#### 2.2.4. Relevant Channel Beliefs

The term RCB refers to individuals’ assessment of whether a particular information channel can provide useful, unbiased and trustworthy information [9]. It is applied to risk management criteria by Griffin et al. [50], focusing on the perception whether a particular information channel contains information that is most relevant to individual’s seeking and processing behavior. With the emergence of new types of information channels, people rarely consult just one specific information channel. In some studies, RCB is replaced by the theory of planned behavior’s (TPB) behavioral beliefs concept [16]. Kuttschreuter et al. [51] used a segmentation approach to identify consumers’ inclination toward different information channels and found that social media channels do not crowd out traditional mass media and, instead, a social media channel can act as a complementary information channel. Wu et al. [9] also analyzed consumers’ trust in different channels and found that trust in information channels significantly influences purchase intention. Thus, the following hypothesis is proposed:

**Hypothesis 4** **(H4).** *RCB is positively related to information-seeking intention.*

#### 2.2.5. Perceived Information Gathering Capacity

Perceived information gathering capacity (PIGC) refers to the perceived ability and control an individual has to perform information-seeking and processing steps, especially to handle issues requiring more cognitive effort and nonroutine gathering of information [14]. It is something like self-efficacy in Social Cognitive Theory (SCT) [52] and perceived behavioral control (PBC) in TPB [29]. In RISP, PIGC plays a vital role in the information-seeking decision. People with information needs are still required to assess various information options available to them and choose the most accessible channel. Hence, the higher the PIGC, the more perceived behavioral control an individual holds relating to information-seeking. Although PIGC was initially proposed to be positively correlated to information-seeking intention and behavior in RISP, the empirical results have shown mixed evidence [16]. Therefore, the following hypothesis is proposed as:

**Hypothesis 5** **(H5).** *PIGC is positively related to information-seeking intention.*

**Figure 1:**
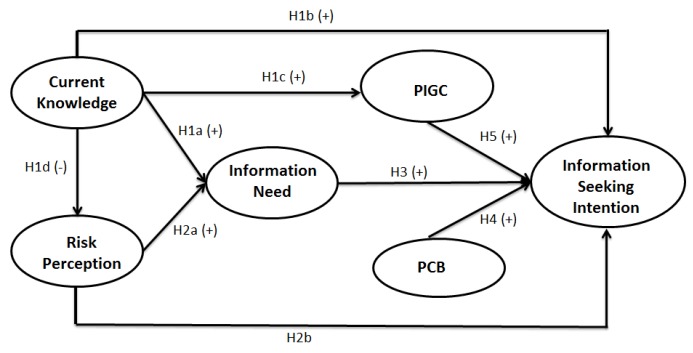
Research framework and research hypotheses (H1–H5) of consumers’ information seeking intention. PCB:Perceivedch annel beliefs; PIGC: Perceived information-gathering capacity. The “+” and “−” signs in brackets denote the direction of the influence.

The research framework is depicted in Figure 1.

## 3. Data and Methodology

### 3.1. Questionnaire Design and Measures

A questionnaire survey was used to collect data. White wine industry is regarded as a main source of food recall of abuse of additive, since lots of additives have been added into the wine in production. In the special inspection project of white wine, lots of white wine have been identified as unqualified product because of addition of additives such as sodium cyclamate. Hence, in this study, we mirrored actual CFDA food recall releases with identical facts and reasons, only the dates and identifying information altered. Us the fictional white wine recall, we intended to explore consumers’ information-seeking intention and the determinants.

The questionnaire included four parts. The first part of the questionnaire introduced the intention of the survey. In the second section the fictional white wine recall is provided. Section three included questions of six constructs of the proposed framework. The last part dealt with general demographic questions of age, gender, education, occupation, and monthly income per capita. In total, 19 items were used to measure six variables. Five items of perceived current knowledge were adapted from Eric [53] and Gearhardtet al. [54]. Four items measuring risk perception and five items measuring PCB were adapted from Chen [55] and Wu et al. [9]. The three items to measure PIGC and three items to measure information-seeking intention were adapted from Griffin et al. [56], Eric [53], and Kahlor [16]. Four items measuring information need were adapted from Huurne and Gutteling [46] and Kellens et al. [15]. Some of the wordings of questions were slightly modified to fit the context of a food recall. All responses were measured on a seven-point Likert scale, with 1 meaning “strongly disagree”, and 7 meaning “strongly agree”.

The questionnaire was first developed in English. It was translated into Chinese with the help of two professors in English and Management. Then the Chinese version was translated back into English by another English professor. The research team compared these three copies and discussed them with professors to ensure that the translation was clear and easily understood since the education level of the participants varied. A pilot survey was conducted in which thirty people were invited to answer the questionnaire. Following the feedback provided by these participants, the questionnaire was modified. The final version is provided in Appendix A.

### 3.2. Procedure and Participants

The survey was conducted using both online and offline methods. The questionnaire was posted on Wenjuanxing, an electronic data collection platform popular in China (https://www.wjx.cn/), from 10 January 2017 to 28 February 2017. All the participants anonymously filled in the questionnaires and were assured that their responses would remain confidential. At the same time, printed questionnaires were distributed to consumers in supermarkets and convenience stores by undergraduates in their hometowns, taking advantage of the winter vacation time. Bonuses and gifts were provided to the participants. In the online survey, red envelopes with a random amount (a kind of bonus provided by the research team via the bonus mechanism in the survey platform) were provided once the participant finished and submitted the questionnaire. Small gifts like bookmarks and keychains were given to the participants in the offline survey as well. A total of 957 completed questionnaires were received, 643 online and 314 offline. Questionnaires were checked for validity, and those missing values for the primary variables and those with identical answers in five successive questions were deleted. This resulted in 631 usable questionnaires for further analysis. 

Since the data were collected from participants in two ways, potential structural differences may exist here. We first tested whether the data followed a normal distribution; the result demonstrated that the skewness values of all items ranged between −0.472 and 0.75, while the kurtosis values were between −0.70 and 0.084. Hence, the data follow a normal distribution. Then *t* test was employed and the result showed that the data collection method did not significantly affect the results and so the combined data set is eligible for further analysis. Non-response bias may exist in questionnaire surveys [57] so we compared the demographic characteristics of early and late respondents in the sample. The results showed that no significant differences existed and so non-response bias was not severe in this study. 

Of all the respondents, approximately 54% were female, and most people (86%) were between 15 and 54 years old. Most respondents had finished senior high school or below (62.80%) and 37.20% had an education level of associate degree or above. As for monthly income, half of the respondents earned less than ¥4000 per capita. A χ^2^ test was employed to estimate whether the sample could represent the general distribution of China’s population. The results reveal that the distribution percentages of the characteristics of gender and age in the sample are consistent with those of the population. For the characteristics of education and income, the official data available are from 2010 and are not suitable for comparison with the data of the sample from 2017, although generally, the sample is consistent with the population to some extent. A summary of the demographic data of the respondents is shown in Table 1.

## 4. Data Analysis and Results

Common method bias (CMB) should be considered since all the data were collected from a single source at the same time. CMB is regarded as an ordinary phenomenon in self-reported questionnaire research [58,59]. Hence, Harman’s one-factor test was employed, and the results indicated that all the 19 measurement items were divided into three constructs with eigenvalues higher than 1. These three constructs account for 67.04% of the variance, with the first construct accounting for 27.20% which is lower than the threshold of 30% [60]. This result indicates that CMB was not serious in this case. Based on the analyses mentioned above, the dataset was appropriate for further analysis.

Partial Least Squares Structural Equation Modeling (PLS-SEM) was employed since it is particularly useful for testing complex models and is preferable to covariance-based methods for early-stage theory testing models [61,62]. Here, we used Smart-PLS 2.0 and SPSS 23.0 to conduct the analyses. Two models, i.e., the measurement model and the structural model, were tested successively.

### 4.1. Measurement Model Testing

Following Anderson and Gerbing’s [63] two-step analysis, confirmatory factor analysis (CFA) was conducted to examine the reliability and validity. One items (PCB4) was deleted because their factor loadings are less than the threshold of 0.70. As shown in Table 2, the loadings of all items ranged between 0.73 and 0.94 so all were higher than the threshold of 0.7. The internal consistency of the indicators for each construct was analyzed by construct reliability. Values of Cronbach’s alpha ranged from 0.78 to 0.92 and the composite reliability values were between 0.86 and 0.94, all higher than the recommended benchmark of 0.7 [64]. Construct validity employs the measurements of convergent validity and discriminant validity. Convergent validity refers to the extent two or more measures are related to each other [65]. Here, average variance extracted (AVE) was used to test for convergent validity. The results showed that all the AVE scores were between 0.61 and 0.85, indicating an excellent result [61]. Discriminant validity was tested by comparing the square roots of AVEs and the correlations among constructs, by the criteria that the former should be higher than the latter [65]. The results showed that all the square roots of AVEs for each construct were greater than the correlations among constructs (Table 3). Furthermore, the bivariate correlations ranged from 0.15 to 0.65, all less than the problematic level of 0.70 [55], indicating no severe multicollinearity existed. Based on the analyses mentioned above, the measurement model fitted the data well and could be used for further estimation.

### 4.2. Structural Model Testing

Smart-PLS 2.0 was used to conduct the structural model test. Here we ran the bootstrapping algorithm with a sample set of 3000 to get the significance of the paths. As shown in Table 4, our results indicate that across the two models, the independent variables had significant and direct effects. It was indicated that current knowledge had a significantly positive relationship with information need (H1a, β = 0.36, *p* < 0.001), information-seeking intention (H1b, β = 0.13, *p* < 0.05), and PIGC (H1c, β = 0.48, *p* < 0.001), supporting H1a, H1b, and H1c. Considering the correlation between current knowledge and risk perception, the result was significant (β = 0.40, *p* < 0.0001) but in the opposite direction proposed in H1d. Hence, H1d was not supported. Furthermore, the risk perception, information need, and PCB, all were found to be positively related to the seeking intention, thus supporting H2b, H3 and H4. However, PIGC was found to influence seeking intention (H5, β = −0.06, *p* < 0.05), but in the opposite direction proposed in H5, hence, H5 was not supported. Furthermore, we tested the mediating effects in the model following the principle proposed by [66]. The results show that information need partially mediates the relationship between current knowledge, risk perception, and information-seeking intention. Moreover, risk perception fully mediates the relationship between current knowledge and information need.

Next, we examined the PLS-SEM model fit. Following Hair et al. [67], two indicators of the quality of the model adjustment were estimated: predictive validity (Q^2^) and effect size (f^2^). Here, we used Stone–Geisser indicators (Q^2^) to evaluate how much the model approaches the expected fitness, with a threshold of Q2 values greater than zero [67]. The results shown that values of Q^2^ ranged from 0.61 to 0.84, all higher than the recommended threshold of zero. F^2^ measured by Cohen’s indicator was used to measure how useful each construct is for the adjusted model. Values of 0.02, 0.15, and 0.35 are considered levels of small, medium, and large respectively [67]. All the values of f^2^ ranged from 0.06 to 0.19, representing levels of small and medium. Furthermore, the Global quality of the adjusted model was used to estimate the goodness of Fit (GoF) of the model. The GoF value of 0.47 was higher than the recommended threshold of 0.36, indicating the model had an adequate adjustment [68,69]. 

Next, we examined the PLS-SEM model fit. Following Hair et al. [67], two indicators of the quality of the model adjustment were estimated: predictive validity (Q^2^) and effect size (f^2^). Here, we used Stone–Geisser indicators (Q^2^) to evaluate how much the model approaches the expected fitness, with a threshold of Q2 values greater than zero [67]. Values of Q^2^ are all higher than the recommended threshold of zero. F^2^ measured by Cohen’s indicator was used to measure how useful each construct is for the adjusted model. Values of 0.02, 0.15, and 0.35 are considered levels of small, medium, and large respectively [67]. Here, f^2^ ranged from 0.06 to 0.19, representing levels of small and medium. Furthermore, the Global quality of the adjusted model was used to estimate the goodness of Fit (GoF) of the model. The GoF value of 0.47 was higher than the recommended threshold of 0.36, indicating the model had an adequate adjustment [68,69]. 

Values of adjusted *R*-squared are exhibited in Table 2. To summarize, the overall model accounted for up to 47% of the variance in information-seeking. According to the meta-analysis of the RISP model, the estimated adjusted variance explained values for information-seeking were between 0.10 and 0.72, with a median of 0.52 [32]. Hence, the explanatory power of this model is almost in accordance with the median level, which means it has good explanatory power in predicting individuals’ intention to conduct information-seeking.

## 5. Discussion

The current study explores the determinants of consumers’ information-seeking intention based on an augmented RISP model. The main findings are discussed as follows. In this study, current knowledge exhibited a critical role in predicting information need, PIGC, and information-seeking intention in the proposed direction. People who perceive themselves to have more knowledge about food additives need more information, have a higher level of perceived competence and have more intention to seek more information. This finding is consistent with previous studies in the domain of food safety [36,37], indicating that more knowledge can help the assessment of information possessed and needed, as well as provide baseline knowledge for accessing further data. However, current knowledge demonstrated a positive correlation with risk perception, which contradicts H1d as well as the results of previous studies [70,71]. Klerck and Sweeney [71] confirmed that both objective and subjective knowledge reduces people’s risk perception in the adoption of genetically modified foods. A possible explanation of our finding may be stated as follows. In China, the abuse of food additives has become the most common type of food safety incident and a major public concern [24]. In the Food Safety Risk Assessment Report of Shenzhen Municipality, the abuse of food additives was listed as the first of the top ten factors in food safety incidents in Shenzhen, a megacity in southern China. Since so many kinds of food additives are used in food production, people are unfamiliar with the names, chemical composition, and effects of these additives. Hence, it is hard for the public to identify and remove the additive from the food consumed, resulting in higher health risk perception [72]. When people find that a food safety problem is challenging to prevent or eliminate through individual effort, their fear increases [48]. As more food recalls due to abuse of additives occur, more information concerning food recall and specific kinds of food additives are released to the public. Since most of the information released in food recall announcements is negative information about food safety, the information leads to higher perceived risk. Hence, knowledgeable residents also observed higher perceived risks. This study’s significantly positive effects of knowledge over information need and information-seeking intention are consistent with [43], which indicated that more knowledge contributes to higher risk judgment and understanding capacity when making information-seeking and processing [73,74].

Information need or information insufficiency is a critical motive for information-seeking and processing, occupying the central part of the models of RISP [11], framework of Risk Information Seeking (FRIS) [44], PRISM [13], and Yang et al. [32]. Consumers with high levels of information insufficiency would prefer to seek more information to narrow the knowledge gap. Since varieties of food additives and illicit substances are used in food production, consumers generally lack sufficient knowledge in most food recalls due to abuse of additives, which leads to a high level of information need and seeking behavior.

As a crucial factor affecting public food scares, risk perception positively influences information need and information-seeking intention. The results of this study confirm the previous hypothesis proposed in RISP and PRISM [11,13]. The repeated food recalls due to abuse of food additives and illegal use of non-food substances in China has increased the risk perception held by the public. In this circumstance, people tend to seek more knowledge and information to take precautions to avoid consuming foods that are problematic because of abuse of additives and illegal chemicals.

Concerning perceived channel beliefs, this study’s result demonstrated that PCB significantly and positively influences the seeking intention, agreeing with previous studies in the domain of food safety and risk perception. Lobb et al. [75] revealed that with a high level of perceived trust in the communication channels, people display less likelihood of consuming chicken after an outbreak of avian flu. In the case of China, Wu et al. [9] also analyzed consumers’ trust in different channels and found that trust in information channels significantly influenced purchase intention. In the case of food recalls due to abuse of food additives, the specified recall information and knowledge were released and spread through diversified channels. People need to determine which information channels are trustworthy and base their decisions on information released through these channels accordingly. The higher the level of consumer trust in the available information channels, the higher the likelihood for them to conduct seeking behavior [75]. 

However, PIGC demonstrates a negative correlation with risk perception, which contradicts H5. Hence, H5 was not supported. This result conforms to the studies of Kahlor [12] and Yang [76]. This is not surprising, since respondents who perceived themselves competent in gathering information are less likely to seek concerned information. A possible reason may be explained as follows. People with high self-perceived personal control over the recall situation might delay forming the seeking intention and conduct behavior because they think that they can do it whenever it is necessary and urgent. Hence, they would not report seeking intention in the context of common issues. Of the three levels of food recall, the third level mainly concerns the mislabeling and packaging, which is not perceived as immediate harm to public’s health. Hence, people felt less urgent to evaluate the potential harm, leading to less seeking intention. Therefore, people with high efficacy and personal control would tend to shy away from information-seeking. 

## 6. Conclusions, Implications and Limitations

Considering the severe food safety situation in China, food recall has been regarded as a systematic and effective solution. In this paper, an augmented conceptual framework was proposed based on RISP. The survey data from 631 respondents were collected through online and offline methods in China. PLS-SEM analysis was employed to analyze the data.

The study has following findings. Firstly, the empirical results revealed that current knowledge and perceived risk positively influence information need, perceived channel beliefs, and information need. All these results support the application of the augmented RISP model and conform to the results of previous studies. Thus, our study results enrich the information communication research in the domain of food safety and food recalls. Secondly and most importantly, we tested the central role of PIGC in the augmented RISP model. The results show that current knowledge influences PIGC, and PIGC negatively influences information-seeking intention. Moreover, we analyzed the direct effect of PIGC on information-seeking intention, effects which were concluded to be under-explored in previous studies [32,77]. The results show that PIGC exerts a negative direct effect, contradicting to the original hypotheses in the RISP model and the assumptions in this paper. Thirdly, the relationship between current knowledge and perceived risk was shown to be positive, which was contrary to our hypothesis. This finding motivates further research in risk analysis in the context of food safety in the domain of food recall.

These findings also provide some practical implications. Food safety is always a hot topic in China, and food additives are listed at the top of factors of food safety risks perceived as perceived by the public. The government, food manufacturers, and retailers should pay attention to these determinants of consumers’ information-seeking intention. Firstly, the concerned stakeholders should provide timely and accurate recall information and knowledge to the public. As indicated in the study, current knowledge is a central factor in the proposed model; current knowledge positively relates to PIGC, risk perception, seeking need, and seeking intention. Hence, food safety education is an important factor when dealing with food recalls. Popular science education and recall communication concerning food recall and food safety should be provided by the stakeholders. Due to the strong technology involved in food recall, the lack of clarity and media coverage is of great concern [78]. On the one hand, the CFDA and its subordinates should provide basic knowledge of food recalls and food safety via the official websites and social media. It is particularly important for the government to provide detailed information when the recalled food involves great potential harm and raises a high level of public fear. On the other hand, besides the required recall announcements, the involved manufacturers and retailers should provide detailed information about the cause and the potential harm, as well as the countermeasures to be taken in regard to the recalled foods [79]. Hence, the recalling manufacturers and retailers can mitigate the possible damage and save their corporate reputations by providing helpful information and services. For example, supplemental advertising provided by recalling manufacturers has been found to be of benefit if it conveys risks of consumption and recommendations to the consumers clearly [18]. Secondly, the communication channels should be extended. As indicated in this study, PIGC has a significant direct influence on information-seeking intention. Hence, the stakeholders should increase the “ability to access” and improve the externally focused perceived control [13,16,77]. The release of food recall announcements currently relies mainly on mass media (local newspapers, TVs, and broadcasting) and internet-based social media (official websites plus WeChat and Weibo subscriptions of the CFDA system, manufacturers, and other parties) [77]. However, as a necessity in daily life, food purchasing and consumption is conducted by all sorts of people of different ages. People who are old and have little education may find it hard to access internet-based information and social media. Considering that recalled food may harm the elderly more than the young, easily accessible channels should be designed for the elderly. Alongside the traditional mass media (television and media reports), point-of-purchase information is regarded as an effective method [79]. The in-store location of food recall information was preferred by respondents in two successive surveys in the USA [79]. The notice should be posted either on the shelf near where the product was purchased or at the store entrance. The very public notice serves as a reminder to the public to check whether their past purchase is related to a recall, especially for the elderly. Some capable supermarkets can even print the recall information on the receipt for elderly consumers to check whether they have bought the recalled food when they return home [79]. Simply put, improving the accessibility and feasibility of food recall information can lead to better food safety management.

Alongside the findings and applications mentioned above, it is important to highlight the limitations of the current research. Firstly, this research only investigated individuals’ information-seeking intention rather than actual seeking behavior. Generally, there is a gap between behavioral intention and actual behavior [29,80]. Also, in the RISP model, these antecedents will eventually influence heuristic and systematic information processing behaviors [32]. Hence, the researchers should further analyze individuals’ actual seeking behavior by using interviews and other experimental methods to determine actual behavior. Secondly, individual characteristics and relevant hazard experience were predicted to be antecedents in risk information management theories [32]. Gender, age, education level, and relevant hazard experience can influence risk perception and motivation as well, which will further influence information-seeking and processing. So, all these individual characteristics should be included to augment the model in future studies. Lastly, though we have explored the direct effect of PIGC and PCB, we do not specifically distinguish different types of information channels and consumers’ trust in the specific channels. Further studies on categorized channels and trust in the information conveyed by these channels would provide interesting and usable information to perfect communication systems and improve the efficiency of the management of food safety and food recall.

## Figures and Tables

**Table 1:** Demographic profile of respondents.

Demographics	Frequency	Percentage (%)
Gender		
0. Male	290	46
1. Female	341	54
Age		
1. under 15	26	4.1
2. 15–34	193	30.6
3. 35–54	355	56.3
4. 55 and above	51	9.1
Education level		
1. Preliminary school or below	15	2.4
2. Senior high school or below	381	60.4
3. Associate degree or bachelor degree	123	19.5
4. Master’s degree or PhD	112	17.7
Income		
1. Less than ¥2000	124	19.7
2. ¥2001–¥4000	206	32.6
3. ¥4001–¥6000	125	19.8
4. ¥6001–¥8000	124	19.7
5. More than ¥8001	52	8.2
Total	631	100

**Table 2:** Results of the confirmatory analysis.

Construct	Item	Factor Loading	Cronbach’s Alpha	Composite Reliability	AVE	*R*-Squared
Perceived information-gathering capacity (PIGC)	PIGC1	0.92	0.91	0.94	0.84	0.23
	PIGC2	0.94	-	-	-	-
	PIGC3	0.89	-	-	-	-
Information-seeking intention (INT)	INT1	0.90	0.91	0.91	0.77	0.47
	INT2	0.90	-	-	-	-
	INT3	0.82	-		-	-
Current Knowledge (Know)	Know1	0.8	0.92	0.94	0.75	
	Know2	0.89	-	-	-	-
	Know3	0.89	-	-	-	-
	Know4	0.88	-	-	-	-
	Know5	0.86	-	-	-	-
Information need (Need)	Need1	0.73	0.78	0.86	0.61	0.39
	Need2	0.82	-	-	-	-
	Need3	0.77	-	-	-	-
	Need4	0.79	-	-	-	-
Perceived channel beliefs (PCB)	PCB1	0.81	0.85	0.89	0.62	-
	PCB2	0.82	-	-	-	-
	PCB3	0.78	-	-	-	-
Risk perception (Risk)	Risk1	0.92	0.90	0.93	0.77	0.16
	Risk2	0.9	-	-	-	-
	Risk3	0.82	-	-	-	-
	Risk4	0.86	-	-	-	-

AVE: Average variance extracted.

**Table 3:** Means, standard deviation, and correlations.

Construct	Mean	SD	PIGC	INT	Know	Need	PCB	Risk
PIGC	3.73	1.44	**0.84**	-	-	-	-	-
INT	5.06	0.94	0.20 **	**0.72**	-	-	-	-
Know	5.02	0.97	0.48 **	0.41 **	**0.75**	-	-	-
Need	4.38	5.11	0.30 **	0.48 **	0.51 **	**0.61**	-	-
PCB	5.52	0.77	0.15 **	0.48 **	0.37 **	0.42	**0.62**	-
Risk	5.19	0.93	0.30 **	0.65 **	0.40 **	0.53 **	0.53 **	**0.77**

Means are measured based on average factor scores; SD: Standard deviation. The diagonal (bold) elements are the square roots of AVEs, and the off-diagonal figures are the correlations among constructs. **: significance at or below the 0.01 level.

**Table 4:** Path coefficients of the structural model.

Path	Coefficient	*t* Statistics	*t* Statistics	Hypothesis	Results
Know -> Need	0.36 ***	8.56	8.56	H1a	Supported
Know -> INT	0.13 *	2.45	2.45	H1b	Supported
Know -> PIGC	0.48 ***	15.93	15.93	H1c	Supported
Know -> Risk	0.40 ***	9.08	9.08	H1d	Not supported
Risk -> Need	0.39 ***	8.47	8.47	H2a	Supported
Risk -> INT	0.48 ***	10.66	10.60	H2b	Supported
Need -> INT	0.13 *	2.51	2.51	H3	Supported
PCB -> INT	0.13 **	3.10	3.10	H4	Supported
PIGC -> INT	−0.06 *	2.14	2.14	H5	Not supported

* *p* < 0.05; ** *p* < 0.01; *** *p* < 0.001.

## Appendix A

**Table A1:** Constructs of the proposed model.

Construct	No.	Items
INT	Int1	I plan to seek more information about abuse of additive (e.g. sodium cyclamate) involved in white wine recall issues.
	Int2	I intend to find out more about abuse of additive (e.g. sodium cyclamate) used in white wine recall issues.
	Int3	In the future, I will try to seek as much information as I can about abuse of additives used in other types of food.
PIGC	If I want to get more information about food recall issue and concerned food additive,	
	PIGC1	I could obtain information from the CFDA and its sub-ordinates.
	PIGC2	I could obtain information from the social media.
	PIGC3	I will readily take time to gather any additional information I might need.
Need	Need1	I need information about the abuse of food additive (e.g. sodium cyclamate) involved in the white wine recall.
	Need2	I need lots of information about the possible effects of food additive (e.g. sodium cyclamate) involved in the white wine recall.
	Need3	I need to know the latest news related to white wine recall issue of abuse additive (e.g. sodium cyclamate).
	Need4	I want the government to provide more information about different types of food additives.
Know	Know1	I know what food additives (e.g. sodium cyclamate) are.
	Know2	I know the harm of food additives (e.g. sodium cyclamate).
	Know3	I Know the intended use of food additives (e.g. sodium cyclamate).
	Know4	I know the effect of over use of legal food additive (e.g. sodium cyclamate).
	Know5	I know the effect of use of illegal food additive (e.g. sodium cyclamate).
Risk	Risk1	Consuming white wine with abuse of additive (e.g. sodium cyclamate) is dangerous to me.
	Risk2	Consuming white wine with abuse of additive (e.g. sodium cyclamate) will seriously influence my health.
	Risk3	Consuming white wine with abuse of additive (e.g. sodium cyclamate) will bring loss to our property.
	Risk4	Effects of additive (e.g. sodium cyclamate) found in the recalled white wine will make me unhappy when I think of wine-drinking.
PCB	Do you believe you can obtain accurate information about the food recall issue and concerned food additive through the following channels?	
	PCB1	Website and official account (in Wechat and Weibo) of governments, especially the China Food and Drug Administration, and the sub-Bureaus.
	PCB2	Social network software (i.e., WeChat, QQ, microblog, and others.).
	PCB3	Peers (i.e., relative, friend, and colleague).
	PCB4	Manufactures and retailers of the recalled food.

PCB4 were deleted because their factor loadings were below 0.70.
